# Genetic Variability for Grain Components Related to Nutritional Quality in Spelt and Common Wheat

**DOI:** 10.1021/acs.jafc.3c02365

**Published:** 2023-07-03

**Authors:** Ana Belén Huertas-García, Facundo Tabbita, Juan B. Alvarez, Josefina C. Sillero, M. Itria Ibba, Marianna Rakszegi, Carlos Guzmán

**Affiliations:** † Departamento de Genética, Escuela Técnica Superior de Ingeniería Agronómica y de Montes, Edificio Gregor Mendel, Campus de Rabanales, Universidad de Córdoba, CeiA3, Córdoba ES-14071, Spain; ‡ Instituto de Recursos Biológicos, Instituto Nacional de Tecnología Agropecuaria (INTA), N. Repetto y los Reseros s/n, Hurlingham 1686, Buenos Aires, Argentina; § IFAPA Alameda del Obispo, Avenida Menéndez Pidal s/n, Cordoba 14004, Spain; ∥ Global Wheat Program, International Maize and Wheat Improvement Center (CIMMYT), Apdo Postal 6-641 Mexico DF, Mexico; ⊥ Agricultural Institute, Centre for Agricultural Research, Brunszvik u. 2, Martonvásár 2462, Hungary

**Keywords:** ancient wheats, nutritional quality, fiber, phytic acid, micronutrients

## Abstract

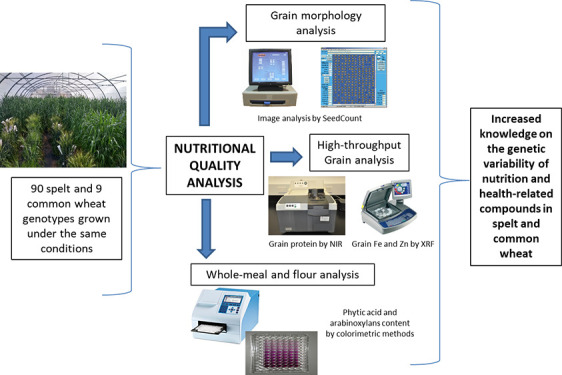

Spelt (*Triticum aestivum ssp. spelta*) is part of the so-called ancient wheats. These types of wheats are experiencing a revival as they have been proposed to be healthier than conventional wheat. However, the given healthier condition of spelt is not substantiated by solid scientific evidence. The objective of this study was to analyze the genetic variability for several grain components, related to nutritional quality (arabinoxylans, micronutrients, phytic acid) in a set of spelt and common wheat genotypes to determinate if spelt is potentially healthier than common wheat. The results obtained indicated that within the compared species, there is a significant variation in the nutritional compounds, and it is not truthful and accurate to state that one species is healthier than the other. Within both groups, genotypes showing outstanding values for some traits were detected, which could be used in breeding programs to develop new wheat cultivars with good agronomic performance and nutritional quality.

## Introduction

Wheat is the world’s most widely grown crop, occupying 221 million hectares and accounting for a quarter of total cereal production, with 771 million tons produced in 2021.^1^ It is the staple food for about 40% of the world’s population providing between 20 and 50% of total caloric intake in temperate countries. However, wheat is more than a source of calories as its grain contains significant amounts of other nutrients essential for correct physical and mental development and healthy life.^2^ In fact, scientific evidence shows that regular consumption of wheat-based foods, preferably whole grains, provides health benefits such as reduced risks of obesity or overweight, type 2 diabetes, blood pressure, and some cancers.^3^

The wheat grain is rich in protein, showing around 12–14% on average. Because of its widespread consumption, it accounts for almost 20% of total global dietary protein. In addition to its nutritional importance, a large part of the wheat grain protein is composed of the gluten proteins, which are responsible for the unique properties of the wheat dough that allow the preparation of hundreds of different foods appealing to consumers. Because of this, grain protein content (GPC) is of great interest in wheat genetic improvement programs, which generally aim to increase it, although that is difficult due to the negative relationship of this trait with grain yield. Furthermore, several micronutrients important for human nutrition such as iron (Fe) and zinc (Zn) are also present in the wheat grain in significant amounts, contributing 44 and 25% of the daily intake in developed countries, respectively.^2^ This could probably be higher in developing countries where wheat is the main staple food. In these last regions, more than 2 billion people suffer from a certain degree of micronutrient deficiency (mainly Fe, Zn, or vitamin A),^4^ which has made breeding programs include these traits in their breeding pipelines to alleviate this problem.^5^ Related to this, phytic acid (PA) is another important molecule that acts as a primary phosphate reservoir in the seeds. However, due to its ability to chelate micronutrients such as Fe and Zn, it is often considered as an antinutrient, and thus, ideally, the new wheat cultivars developed to fight against malnutrition should have reduced PA content in the grain. However, PA has also been associated with the prevention of major health risks such as the cardiovascular diseases or cancer.^6^

Dietary fiber is another important component of wheat grain, being wheat products are one of the main sources of this bioactive component, accounting for approximately 40% of dietary fiber intake in the UK.^7^ The main types of dietary fibers in wheat grains are β-glucans and arabinoxylans (AXs), being the latter by far the most abundant.^8^ AXs are usually divided into two classes, depending on whether they are water extractable (WE-AXs) or nonextractable (WU-AXs). Both types have different effects on human health^9^ and processing and end-use quality.^10^ As in the case of micronutrients, breeding programs are starting to target these grain components as well, in order to develop novel wheat cultivars with enhanced health properties.^11,12^

Among the wheat species currently grown, bread or common wheat (*Triticum aestivum ssp. aestivum*) is by far the most important species, covering around 95% of the total wheat cultivated area. However, the need for more sustainable agriculture and crop diversification has led to a renewed interest in other wheat species such as spelt wheat (*T. aestivum ssp. spelta*).^13^ Spelt wheat is hulled wheat that forms part of the so-called ancient wheats. These types of wheats were important in the past but were replaced by modern wheat cultivars due to their reduced agronomic performance. Probably, the most important reason for the revival of this species is that it has been proposed to be a better source of bioactive components than conventional and hence suitable for producing healthier and more ‘natural’ food products. Although a number of studies on spelt grain composition have revealed significant variation,^14,15^ the given healthier condition of spelt compared to modern wheat is not substantiated by solid scientific evidence. There are a limited number of systematic studies on the detailed composition of spelt versus currently grown common wheat cultivars, and it would be useful to know more about spelt diversity for nutritional grain components and how it differs from common wheat.^9,16^ In addition to the interest in spelt as a crop, the useful genetic variation found in this species could also be used as a source for the development of more nutritious common wheat.^17^

The objective of this study was to analyze the genetic variability for several grain components related to nutritional quality in a set of spelt and modern common wheat genotypes to determinate if (1) spelt has a better grain composition from the nutrition and health point of view than modern common wheat and (2) to identify superior genotypes that could be used in breeding programs to develop high yielding adapted novel cultivars with high nutritional quality.

## Materials and Methods

### Plant Material and Field Trials

For this study, 89 Spanish spelt wheat accessions and 10 modern common wheat cultivars were used (Table S1). These 89 spelt accessions were originally provided by the National Small Grains Collection (USDA, USA) and Centro de Recursos Fitogeneticos (INIA, Spain) and were purified and analyzed in previous studies showing significant variability for different traits.^18,19^ These accessions are traditional landraces and have not been hybridized with modern wheat. Of the 10 modern cultivars, nine of them were commercial Spanish common wheat cultivars commonly grown in Andalusia (South of Spain) and that represent well the diversity found in farmers’ fields. The other cultivar was Anna Maria, a modern spelt wheat cultivar released in 2018 and derived of hybridization of spelt with modern common wheat.

These 99 wheat genotypes were planted in 0.13 m^2^ plots with two replicates in a randomized complete block design under full drip irrigation during 2019–2020 and 2020–2021 crop seasons in Cordoba (Andalusia, Spain). Weed, diseases, and insects were all well controlled. Nitrogen fertilizer was applied (preplanting) at a rate of 50 kg of N/ha and at tillering 150 additional units of N and enough amount of micronutrients (including Fe and Zn) were applied.

### Grain Quality Traits

Thousand kernel weight (TKW, g) and test weight (TW, kg/Hl) were obtained using the SeedCount SC5000 digital imaging system (Next Instruments, Australia). GPC (%) was determined by near-infrared spectroscopy (NIR Systems 6500, Foss Denmark) calibrated based on AACC official methods 39-10.01, 55-30.01, and 46-11.02, respectively.^20^

An energy-dispersive X-ray fluorescence spectrometry instrument (EDXRF, Oxford Instruments, Abingdon, UK) was used to determine Fe (mg/kg) and Zn content (mg/kg) in grains. A Megazyme scale-down protocol was used to determine the concentration of PA in whole-meal flour,^21^ obtained with a Udy Cyclone-type mill. The molar ratios of PA:iron (PA:Fe) and PA:zinc (PA:Zn) were also calculated. WE-AXS and total AXS (TOT-AX) were determined in both whole-meal and refined flour (obtained by milling in a Brabender Quadrumat Senior Mill) using the colorimetric method reported by Hernández-Espinosa et al.^21^ Because of the grain amount needed, the field repetitions were mixed to produce the refined flour. So, one unique data per genotype and year was obtained for TOT-AX and WE-AX in refined flour. The amount of mixed-linkage β-glucans in whole-meal flour samples was determined using a Megazyme kit (Megazyme, Bray, Ireland) according to AACC 32-23.01 standard method.^20^ β-glucans content was only determined in 14 of the genotypes (11 spelt accessions and three modern wheat cultivars) of the study due to a lack of enough grain in the rest of the samples to perform the analysis. Duplicate analyses were carried out on each sample.

### Statistical Methods

A multivariate analysis with all quality traits measured was performed by a principal component analysis using the covariance matrix between all genotypes. The comparison between both species sets was carried out for each trait analyzed by the Student *t*-test.

For spelt set, data were analyzed by an analysis of variance (ANOVA) using genotype, year, and genotype × year as variation sources. The means were compared by the Tukey method. The differences among the value of each genotype and the mean of the common wheat genotype used as control were used to value the potential of the spelt genotypes for wheat breeding.

Correlation analysis between the measured traits was performed and represented in a matrix indicating significance values. Statistical analyses were carried out using Rstudio (version 4.2.1, Vienna, Austria) and Infostat software (version 29-09-2020, Cordoba, Argentina).

## Results

### Spelt versus Common Wheat

**Figure 1:**
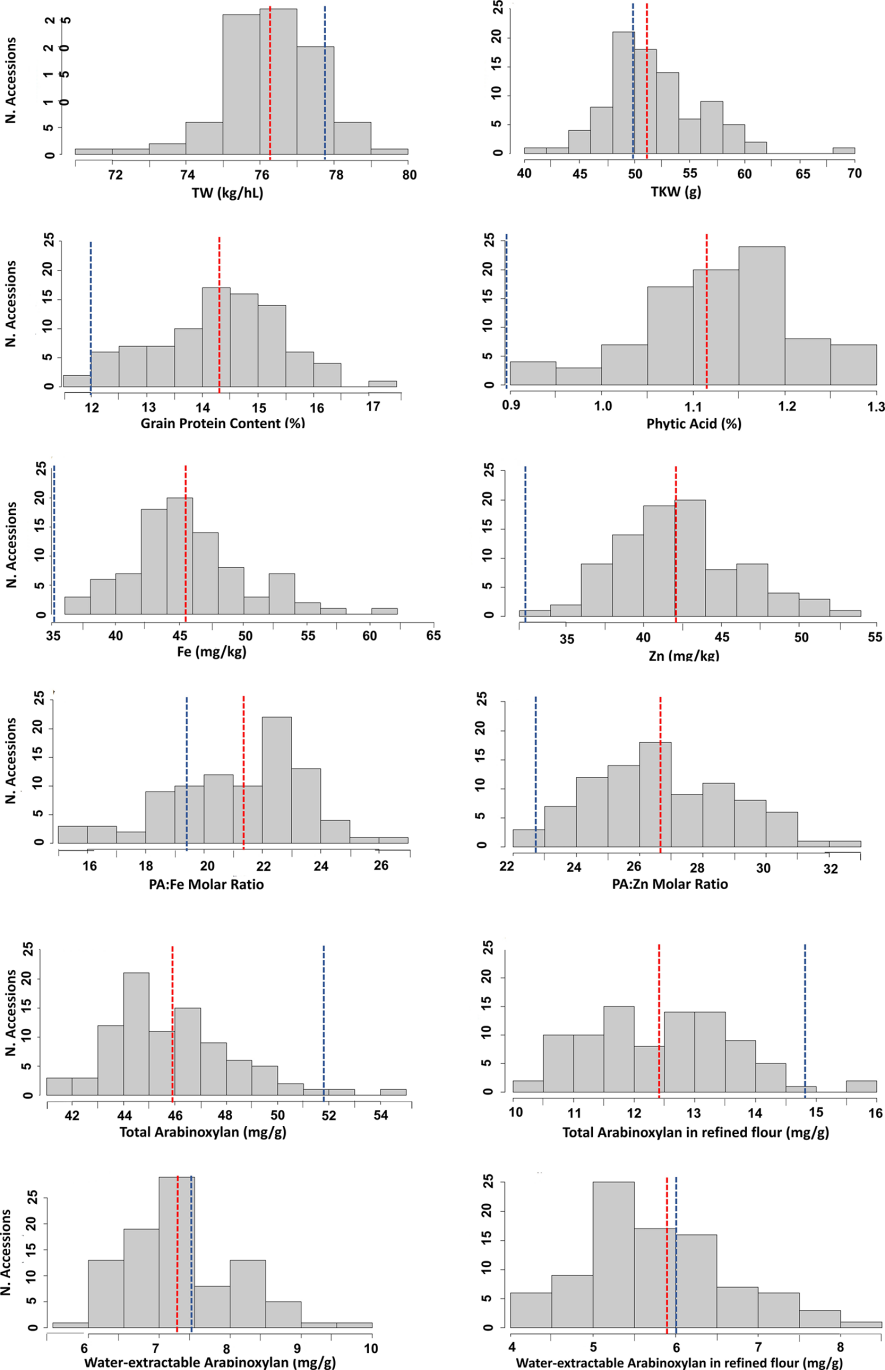
Number of spelt genotypes found in each range of variation for the different quality traits evaluated across the two cropping cycles of the study. Red and blue dots lines indicate the mean value of spelt genotypes and modern common wheat cultivars groups, respectively (averaging genotypes and years).

**Figure 2:**
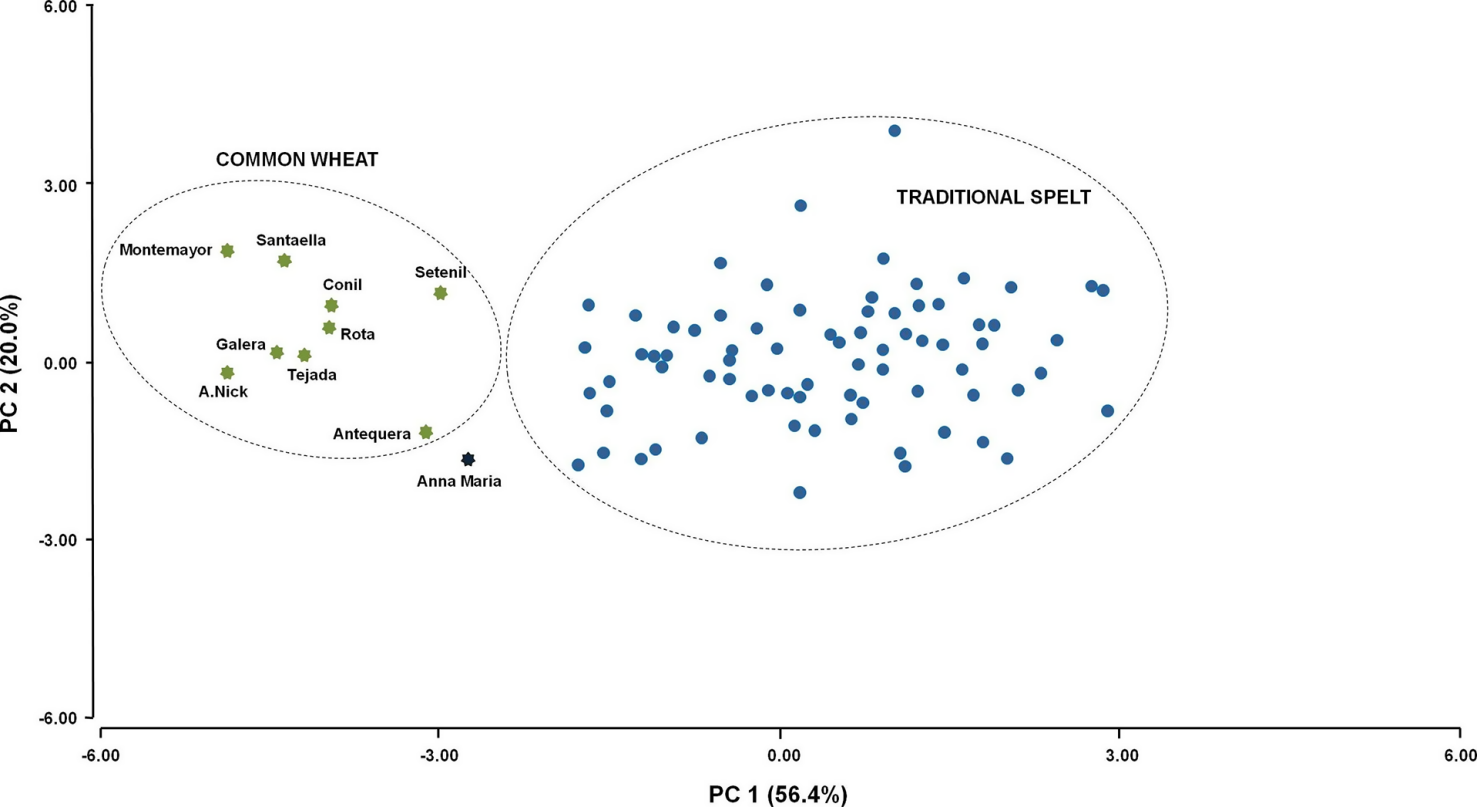
Principal component analysis of the quality traits analyzed. The distribution of the spelt landraces is shown in blue spots and that of the modern common wheat cultivars in green spots. Anna Maria, a modern spelt wheat cultivar, is shown in a black spot.

Diverse quality traits were measured for all materials used in this study; these data are shown in Table S2. For all these traits, the spelt genotypes showed large ranges of variation across 2 years of the study compared with the common wheat cultivars used as controls (Figure 1). The variation between the two data sets (90 spelt genotypes and 9 modern common wheat cultivars) was analyzed by a principal component analysis. Up to 76.4% of the observed variation was determined by PC1 (56.4%) and PC2 (20.0%). These two new variables permitted easily to discriminate between the spelt and modern common wheat genotypes (Figure 2). Furthermore, this analysis showed that cv. Anna Maria, modern spelt wheat, was closer to the modern cultivars group than to the spelt genotypes, indicating that, at least in terms of quality traits, it seemed more similar to modern common wheat cultivars than to the spelt genotypes.

Almost all traits showed significant differences between the two groups (Table 1); for some of them, the differences were small, such as TW, WE-AXs, or β-glucans, while for others such as Fe, Zn, PA, and GPC were large. In terms of protein content, the spelt genotypes showed more than two points of percentage difference. Similarly for micronutrients content, the Fe and Zn grain content was on average 10 mg/kg higher in the spelt genotypes than in the common wheat genotypes; the PA content was also around two-thirds higher in the spelt group, which led to have significantly higher PA:Fe and PA:Zn too. For the traits related to dietary fiber, WE-AXs did not show significant differences between the two groups in both whole-meal and refined flour; TOT-AXs and β-glucans were higher in common modern wheat cultivars (in the case of TOT-AXs in both whole-meal and refined flour).

**Table 1:** Average Values of the Common Wheat and Spelt Groups (Averaging Genotypes and Years) and Result of the *t*-Test Done between both Values^a^

trait	common wheat (*n* = 9) (mean ± s.d.)	spelt wheat (*n* = 90) (mean ± s.d.)	*t*-value
TW (kg/hL)	77.60 ± 2.77	76.25 ± 1.84	3.98^b^
TKW (g)	49.73 ± 6.03	51.68 ± 4.82	–2.25^b^
GPC (%)	11.87 ± 1.14	14.28 ± 1.75	–8.09^b^
Fe (mg/kg)	32.63 ± 3.74	45.63 ± 5.49	–13.89^b^
Zn (mg/kg)	32.79 ± 5.31	42.31 ± 5.55	–9.85^b^
PA (g/100 g)	0.73 ± 0.09	1.12 ± 0.11	–20.39^b^
PA:Fe	19.34 ± 3.66	21.16 ± 3.03	–3.38^b^
PA:Zn	22.49 ± 3.31	26.66 ± 3.34	–7.14^b^
TOT-AX (mg/g)	51.01 ± 3.29	45.90 ± 3.74	7.89^b^
WE-AX (mg/g)	7.56 ± 2.07	7.32 ± 1.97	0.69 ns
TOT-AX (Ref.) (mg/g)	15.32 ± 1.65	12.46 ± 1.95	5.99^b^
WE-AX (Ref.) (mg/g)	6.15 ± 0.93	5.80 ± 1.03	1.40 ns
β-glucan (mg/g)	7.31 ± 0.52	6.64 ± 0.70	2.59^b^

a Fe, iron content; GPC, grain protein content; PA, phytic acid; PA:Fe, phytic acid:iron molar ratio; PA:Zn, phytic acid:zinc molar ratio; Ref., refined flour; TKW, thousand kernel weight; TW, test weight; TOT-AX, total arabinoxylan; WE-AX, water-extractable arabinoxylan; Zn, zinc content.

b Significant at 99.9 and 95%; ns, not significant.

### Variation of Grain Quality Parameters in Spelt

ANOVA was carried out to identify what factors had a larger contribution to the variation found for the quality traits in the spelt set (Table 2). In all cases except for WE-AXs, the genotype was by far the most important factor, explaining a larger percentage of the variation. On average, the genotype explained 51.1% of the variation of the different analyzed traits, with traits such as WE-AXs for which the genotype contribution was smaller (15.6% of the variation) and others such as TKW and Fe content for which it was outstanding (82.1 and 69.1% of the variation, respectively). The genotype × year interaction was in general the second most important factor explaining on average 14.0% of the variation found in all traits. This source of variation was not significant in all cases, with exception of Fe and WE-AXs content. Finally, the year also had a significant effect for all traits except for Fe and PA:Zn content, accounting on average for 11.9% of the registered variation.

**Table 2:** Effects of Genotype, Year, and their Interaction on Quality Traits in Spelt Accessions^a^^,^^b^

trait	genotype sq. sum (%)	year sq. sum (%)	genotype × year sq. sum (%)	error sq. sum (%)	*H*^2^
TW	601.35^c^ (49.41)	143.39^c^ (11.78)	186.41 ns (15.32)	285.85 (23.49)	0.31
TKW	6853.18^c^ (82.16)	500.42^c^ (6.00)	375.34 ns (4.50)	612.27 (7.34)	0.73
GPC	466.72^c^ (42.58)	23.27^c^ (2.12)	156.49 ns (14.28)	449.51 (41.01)	0.28
Fe	7470.74^c^ (69.15)	0.76 ns (0.01)	1483.99^c^ (13.74)	1.848.59 (17.11)	0.56
Zn	5810.53^c^ (52.54)	286.87^c^ (2.59)	1677.08 ns (15.16)	3.284.89 (29.70)	0.37
PA	2.50^c^ (56.03)	0.22^c^ (4.88)	0.69 ns (15.42)	1.06 (23.67)	0.39
PA-Fe	1857.58^c^ (56.50)	46.85^c^ (1.43)	589.49 ns (17.93)	793.76 (24.14)	0.38
PA-Zn	1785.84^c^ (44.91)	0.56 ns (0.01)	806.49 ns (20.28)	1.383.48 (34.79)	0.25
TOT-AX	2103.42^c^ (41.90)	709.81^c^ (14.14)	942.07 ns (18.77)	1.264.41 (25.19)	0.20
WE-AX	218.60^c^ (15.61)	1059.45^c^ (75.65)	64.44^c^ (4.60)	58.03 (4.14)	0.06
β-glucan	19.10^c^ (69.87)	0.24 ns (0.88)	3.25 ns (11.89)	4.74 (17.35)	0.50

a Squares sum (Sq. sum), % of the total squares sum of squares from ANOVA analysis, and broad-sense heritability (*H*^2^) are indicated.

b Fe, iron content; GPC, grain protein content; PA, phytic acid; TKW, thousand kernel weight; TOT-AX, total arabinoxylan; TW, test weight; WE-AX, water-extractable arabinoxylan; Zn, zinc content

c Significant at 99.9 and 95%; ns, not significant.

In the case of TOT-AX and WE-AX in refined flour, the statistical analysis of these traits showed significant differences between both harvest years. On the contrary, the differences among genotypes were only significant for WE-AX.

The β-glucans content was measured on 14 selected spelt genotypes, together with two cultivars of common wheat (cv. Setenil and Tejada). For this trait, the genotypes showed significant differences among them, but the effects of the year and genotype × year interaction were not significant.

### Identification of Superior Spelt Genotypes for Its Use in Breeding

The use of traditional spelt wheat has two possible ways for cereal breeding. On the one hand, it could serve as a donor of specific traits for modern common wheat breeding, by crossing it with modern materials and following the selection of the desirable traits. On the other hand, it could be used as a crop, using the traditional varieties directly in the field or to breed modern spelt cultivars more adapted to current agriculture conditions. Consequently, the traits measured in the current study should be valued according to the concrete breeding finality: introgression in common wheat or development of new spelt cultivars. In this respect, the broad-sense heritability values shown in Table 2 are key for establishing the real possibility of successful transference of these traits to modern wheat by hybridization and posterior selection.

The mean value for each trait for each genotype (averaging years) and the difference in the percentage of this mean value with the average value of the nine modern common wheat cultivars used as checks were calculated (Tables S3a,b). Based on this analysis, it was shown that 11 spelt genotypes, including cv. Anna Maria, present TW values higher than the control mean. Nevertheless, this trait could be influenced by the oblong shape of the spelt grain, and consequently, many spelt genotypes have low values. In any case, the relatively low broad-sense heritability (*H*^2^) value (0.31) suggested that this trait has an important environmental component and its introgression into modern wheat could be difficult. By comparing the different spelt genotypes, several of them (ESP-80, ESP-281, ESP-384, and ESP-387) showed higher values than cv. Anna Maria.

In the case of grain size (TKW), the *H*^2^ value was the highest (0.73), which suggested that this trait was more highly dependent on the genotype and could be transferred successfully to modern wheat. In this respect, up to 58 spelt genotypes had higher values than those of the common wheat controls (49.73 g), with four genotypes exhibiting at least 20% higher TKW values (accessions ESP-36, ESP-244, ESP-245, and ESP-272) than the controls. Among these four spelt lines, the accession ESP-36 had the highest TKW values (TKW = 68 g) which was, however, associated with a low TW value (73.2 kg/Hl, −5.6% compared to the modern common wheat controls). The ESP-244 genotype combined large grains (TKW = 60 g, 22% more than the modern common wheats) with an acceptable TW (76.6 kg/Hl, −1.2% compared to the modern common wheat controls). The spelt cv. Anna Maria presented a low grain size (42.76 g), which opens the possibility of developing new modern spelt cultivars with better grain size by using some of the materials analyzed in this study.

The correlation analysis (Table S4) showed no negative association between TKW and the protein content (GPC), which suggests that the development of materials with large grain size and high protein content could be possible. In fact, several spelt accessions had greater GPC values compared to the average GPC found in common wheat (11.87%). From those, the spelt genotypes ESP-51, ESP-84, and ESP-249 were probably the most interesting, as they combined more than 25% of the GPC found in common wheat controls and also had good grain morphology characteristics (TW and TKW values similar to or higher than that of common wheats), which indicated a good capacity of those genotypes to accumulate protein in large, not shriveled grains.

The previously analyzed traits mostly influence wheat technological quality which is different than nutritional quality, where the presence and amount of the different grain components establishes the differences between superior and inferior genotypes. A large part of the spelt accessions showed a significantly greater concentration of micronutrients (Fe and Zn) than the common wheat controls (32.63 and 32.79 mg/kg) or the spelt cultivar Anna Maria (36.86 and 34.04 mg/kg), with few of them showing outstanding values such as accessions ESP-245 and ESP-288 for Fe content (60 and 57 mg/kg, respectively) or accessions ESP-94 and ESP-252 for Zn content (52 and 51 mg/kg, respectively). The *H*^2^ values of these traits were moderate (Table 2), being higher for Fe content. Many of these spelt accessions with high Fe and Zn content showed at the same time high PA content with a range between 0.906 and 1.289 g/100 g (Table S3a). This last grain component showed high values in cv. Anna Maria (1.038 g/100 g) as well, having only 10 spelt genotypes with lower PA values (Table S3a). Although the PA has been associated with certain health properties,^6^ in relation to the micronutrients, such as Fe or Zn, it shows chelate-forming ability, thus reducing the bioavailability of these micronutrients. Consequently, the accessions with the highest interest for breeding purposes focused on biofortification will be those ones having high oligoelements content and moderate PA content. This could be estimated by the PA:Fe and PA:Zn molar ratios. These values (PA:Fe and PA:Zn) were, in general, high in spelt genotypes with a high-moderate environmental component according to their *H*^2^ values (Table 2). Nevertheless, 17 spelt genotypes exhibited a lower PA:Fe molar ratio than those of the modern common wheat cultivars, but only one genotype (ESP-51) also showed a low PA:Zn molar ratio. It is important to remark that regarding micronutrients content and potential bioavailability, the modern spelt cultivar Anna Maria showed slightly higher Fe and Zn contents than the common wheats but combined with higher PA, which led to larger PA:micronutrients molar ratios.

In terms of fiber content, only a few spelt genotypes had higher values than that of the common wheat controls for total AXs in both whole-meal and refined flour, and the differences were smaller than 11% (Table S3a). There were more remarkable differences for WE-AXs, for which genotypes ESP-224, ESP-227, and ESP-380 had more than 15% of WE-AXs in whole-meal flour and more than 22% in refined flour than in common wheat, which makes them interesting sources of this trait. In any case, the *H*^2^ values of these traits suggested that the effect of the environment is high and, consequently, their transfer to common wheat could be complicated. For β-glucans (for which less spelt and common wheat cultivars were analyzed; Table S3b), only the accession ESP-300 showed a significantly larger amount compared to common wheat, although the difference was not very large (10%) compared to common wheat.

## Discussion

During the last years, changes in the agri-food perception have generated a greater interest in ancient wheats both as crops per se and as donors of useful traits for modern wheat. In the past, these ancient wheats were mainly neglected due to their lower yield, poor adaptation to the agricultural mechanization, and because they required a special dehulling treatment in the mill to separate the chaff from the grain. Currently, their major interest is more related to their use in food.

In this context, spelt, an ancient wheat species neglected during the 20th century, is experiencing a great revival nowadays and is being offered to customers by traditional and gourmet bakeries and many large retailers in Western countries. Probably, an important part of this success is because spelt has been proposed to be a great source of bioactive components and is hence suitable for producing food products with enhanced health benefits. However, there are a limited number of systematic studies on the detailed nutritional quality of spelt wheat compared to common wheat species.^9,14,16^ In general, according to Shewry & Hey,^9^ these comparisons have the problem to have been performed with a limited number of spelt or common wheat cultivars. This could have certainly biased the results, masking the true value of the spelt materials in some cases or attributing their superiority in terms of nutritional quality in others. Certainly, the number of available accessions of spelt or other ancient wheats is smaller compared with common wheat but not so limited as to exclude the possibility of variation within them. Consequently, the evaluation of larger collections is essential, and this has motivated the development of larger studies.^22,23^ In this study, 90 spelt and 9 common wheat genotypes have been compared in terms of grain nutritional components and other quality traits.

The technological quality must be evaluated with caution when the analyzed materials are ancient or old wheats. Changes in baking techniques throughout the last century generated materials adapted to these techniques, far from traditional baking, and consequently, the evaluation of ancient wheat according to modern parameters could not be a good strategy. These characteristics are mainly demanded by millers are physical and chemical characteristics such as TW, TKW, or GPC. Consequently, regardless of the rheological properties, the new materials must present characteristics of interest to the milling industry as a previous step. In this respect, within the accessions evaluated here, some materials presented high values of TKW, an important trait positively related to grain and flour yield.^24^ Other studies have shown more moderate TKW values for spelt, although this depends on the materials evaluated: “pure” spelt (without common wheat introgression) or modern spelt derived from crosses with common wheat.^23,25−27^ In general, these last ones present larger grain size than the pure spelt. However, in this study, genotypes of pure spelt with TKW values larger than 60 g have been found which has not been detected in other studies.^28^ Particularly, the accession ESP-36 had an outstanding high TKW value (68 g), higher than any other value found for this trait in large studies screening thousands of wheat accessions,^29^ which make it interesting to be used in the genetic dissection for this trait or by breeding programs aiming to develop new cultivars with very high grain size. On the contrary, the TW values in the spelt genotypes were low in general. This was expected, as these genotypes were not adapted to the testing area as in previous studies,^22^ and TW reflects well the adaptation of a genotype to a particular environment. Anyway, it was possible to identify spelt genotypes (accessions ESP-92, ESP-250, and ESP-295), combining large grains and TW values as high as the ones of the common wheat checks, which could be useful for wheat breeding programs aiming to develop modern spelt and common wheat cultivars with higher milling quality.

Another important quality trait analyzed that has great importance for the industrial and nutritional quality was GPC, which is in general negatively influenced by grain yield. However, there are cultivars with the ability to combine high values for both traits appreciated by farmers and food industry. For this reason, the search for genotypes with high TKW and GPC values is interesting for the development of new wheat cultivars with potential high grain yield and protein content. To breed competitive high protein cultivars, the accession ESP-216 is probably the most interesting material identified in the current study (37.5 and 2.6% higher GPC and TKW, respectively, than the checks). The accession ESP-94 also had an outstanding GPC (17.2%), but in this case, it could be due to a concentration effect due to the smaller grain size and lower test weight.

Among the nutritional quality traits analyzed in this study, Fe and Zn have gained notable importance in wheat improvement recently. This is mainly because millions of people suffer from some degree of these micronutrient deficiencies in developing countries, which is named as ‘hidden hunger’.^4^ This problem is not unknown in developed countries where access to food is not always parallel to good nutrition. Consequently, the development of modern genotypes with a higher concentration of micronutrients, mainly Fe and Zn, is important for several wheat-growing and wheat-consuming areas. In general, spelt analyzed here showed significantly higher micronutrients content compared to the common wheat checks (32 vs 45 mg/kg for Fe, and 32 vs 42 mg/kg for Zn, respectively), which agrees with previous studies.^30^ In fact, some spelt genotypes have been successfully used for breeding biofortified cultivars, such as Zincol-16, a cultivar developed by CIMMYT-HarvestPlus and released in 2016 in Pakistan.^5^ This cultivar has a great impact on the area (3.5 million metric tons produced in 2021). In particular, some of the spelt genotypes had very good results for the content of these micronutrients, with values higher than 60 mg/kg for Fe (ESP-245) or 51 mg/kg for Zn (ESP-252) and showing good grain sizes at the same time (>52 g for TKW). Spelt genotypes showing high micronutrients content but poor grain characteristics are not very interesting because the high micronutrients content is probably due to a concentration effect associated with low grain yield.^22^ However, all spelt genotypes showed higher PA content than the common wheat checks, which could reduce the bioavailability of Fe and Zn due to its chelate-forming ability.^31^ The same trend was found by Longin et al.^32^ The higher PA values in the spelt group lead to higher PA:Fe or Zn molar ratios than in the common wheat checks in most cases, something in principle negative from the nutritional point of view. The negative impact of PA could be modulated by cultural practices during food preparation: for example, the proofing and fermentation process during baking has been shown to reduce the PA content;^32,33^ therefore, in regions where that practice is applied, a high PA content should have a lower negative impact on Fe and Zn bioavailability. In addition to this, PA has been proven to be a powerful antioxidant with beneficial effects in several diseases such as cancer, increased cholesterol level, and diabetes.^34^ This could recommend its consumption in areas where the supply of micronutrients is guaranteed by a complete and diverse diet. Because of this, some of these spelt materials may be useful in developing wheat with flour carrying more antioxidant compounds for such areas.

Another grain component that is associated with positive effects on health is dietary fiber. In the current study, arabinoxylans (AXs), contrary to the case of the micronutrients described above, were higher concentrated in the common wheat cultivars than in spelt genotypes. This agrees with the finding of Gebruers et al.^8^ and Hernández-Escareño et al.^35^ Nevertheless, the variability of these components was large in spelt accessions, and some superior genotypes were identified, such as ESP-242 (also highlighted before due to its high Zn content). This showed higher values than the common wheat controls in both whole-meal and white flour. This accession was rich in soluble AXs, which is particularly interesting as this fiber type is also related to processing and end-use quality resulting in a positive effect.^36^ The amount of soluble AXs in this accession is far from the best source for this trait described in the literature, cv. Yumai-34 (9 vs 14 mg/g).^8^ Although the data showed low heritability in the current study, in several trials carried out with common wheat,^37−39^ the fiber content has been shown as a character with strong genetic control and high heritability. Consequently, although further studies should be carried out, for the increase of AXs content, due to the complexity of this trait where different genetic regions are involved, it could be interesting to use materials with higher AXs content than cv. Yumai-34 inside the breeding programs.^11^

In summary, the current study suggested that, within the compared species (spelt and common wheat), there is a significant variation in the nutritional compounds, and it is not truthful and accurate to state that one species is healthier than the other. Within both groups, there are promising genotypes for some traits but not combining high values for all traits. Consequently, the consideration of one species, *sensu lato*, as a healthier or more nutritious for food uses, is not acceptable; however, it is true that within these species there are genotypes with outstanding values for particular nutritional traits that could be used as a source of variation in breeding programs.

The data obtained in the current study indicated that some spelt genotypes could be used for improving traits such as grain size and protein, Fe or Zn content. Ideally, these materials could be hybridized with common wheat genotypes of high TW and low PA content, together with high AXs content (for which the spelt group has not shown superiority). These crosses could be used for two different objectives: the development of new common wheat cultivars or, alternatively, new modern spelt cultivars with good agronomic performance and high nutritional quality.

## Supporting Information Available

Plant material used in the study; correlation coefficients among the evaluated traits in spelt (pdf)
Quality traits registered in the study;, average value of each genotype (averaging years) for the different quality traits and difference in percentage with the mean value of the nine modern common wheat cultivars for the same trait, average value of each genotype (averaging years) for the β-glucans content and difference in percentage with the mean value of the nine modern common wheat cultivars for the same trait (xlsx)

## Abbreviations

*H*^2^: Broad-sense heritability
cv.: cultivar
GPC: grain protein content
HMWGs: high molecular weight glutenins
Fe: iron
PA: phytic acid
PA:Fe: molar ratio of phytic acid:iron
PA:Zn: molar ratio of phytic acid:zinc
TKW: thousand kernel weight
TW: test weight
WE-AXs: water extractable arabinoxylans
WU-AXs: nonextractable arabinoxylans
Zn: zinc
