# Supplementary material for: Genetic Variability for Grain Components Related to Nutritional Quality in Spelt and Common Wheat
Source: J Agric Food Chem. 2023 Jul 3;71(28):10598–606. doi: 10.1021/acs.jafc.3c02365 (PMC10360148; doi:10.1021/acs.jafc.3c02365)
Supplement: Supplementary file 1 — jf3c02365_si_001.pdf [file jf3c02365_si_001.pdf]

Supplementary information for:

Genetic variability for grain components related to nutritional quality in spelt and common wheat

Ana Belén Huertas-García[1], Facundo Tabbita[1,2], Juan B. Alvarez[1,*], Josefina C. Sillero[3], M. Itria Ibba[4], Marianna Rakszegi[5] and Carlos Guzmán[1]

[1] Departamento de Genética, Escuela Técnica Superior de Ingeniería Agronómica y de Montes, Edificio Gregor Mendel, Campus de Rabanales, Universidad de Córdoba, CeiA3, ES-14071 Córdoba, Spain;

[2] Instituto Nacional de Tecnología Agropecuaria (INTA), Instituto de Recursos Biológicos, N. Repetto y los Reseros s/n, Hurlingham (1686), Buenos Aires, Argentina

[3] IFAPA Alameda del Obispo, Avenida Menéndez Pidal s/n, 14004 Cordoba, Spain

[4] Global Wheat Program, International Maize and Wheat Improvement Center (CIMMYT), Apdo Postal 6-641, Mexico DF, Mexico

[5] Agricultural Institute, Centre for Agricultural Research, Brunszvik u. 2, 2462 Martonvásár, Hungary

* Corresponding author: jb.alvarez@uco.es

Table S1. Plant material used in the study

Species	Common name	University of Cordoba Accession Number	Germplasm Bank Accession Number
Triticum aestivum ssp. spelta	Spelt	Esp 1	BGE 001947
Triticum aestivum ssp. spelta	Spelt	Esp 5	BGE 002002
Triticum aestivum ssp. spelta	Spelt	Esp 6	BGE 001978
Triticum aestivum ssp. spelta	Spelt	Esp 19	BGE 001990
Triticum aestivum ssp. spelta	Spelt	Esp 25	BGE 012911
Triticum aestivum ssp. spelta	Spelt	Esp 36	BGE 014270
Triticum aestivum ssp. spelta	Spelt	Esp 48	PI 348755
Triticum aestivum ssp. spelta	Spelt	Esp 51	PI 348428
Triticum aestivum ssp. spelta	Spelt	Esp 57	PI 348439
Triticum aestivum ssp. spelta	Spelt	Esp 65	PI 348455
Triticum aestivum ssp. spelta	Spelt	Esp 68	PI 348458
Triticum aestivum ssp. spelta	Spelt	Esp 72	PI 348462
Triticum aestivum ssp. spelta	Spelt	Esp 73	PI 348463
Triticum aestivum ssp. spelta	Spelt	Esp 74	PI 348465
Triticum aestivum ssp. spelta	Spelt	Esp 78	PI 348471
Triticum aestivum ssp. spelta	Spelt	Esp 80	PI 348473
Triticum aestivum ssp. spelta	Spelt	Esp 84	PI 348478
Triticum aestivum ssp. spelta	Spelt	Esp 86	PI 348480
Triticum aestivum ssp. spelta	Spelt	Esp 88	PI 348483
Triticum aestivum ssp. spelta	Spelt	Esp 92	PI 348489
Triticum aestivum ssp. spelta	Spelt	Esp 94	PI 348493
Triticum aestivum ssp. spelta	Spelt	Esp 95	PI 348495
Triticum aestivum ssp. spelta	Spelt	Esp 105	PI 348515
Triticum aestivum ssp. spelta	Spelt	Esp 107	Pi 348519
Triticum aestivum ssp. spelta	Spelt	Esp 121	PI 348544
Triticum aestivum ssp. spelta	Spelt	Esp 135	PI 348570
Triticum aestivum ssp. spelta	Spelt	Esp 136	PI 348572
Triticum aestivum ssp. spelta	Spelt	Esp 182	PI 348676
Triticum aestivum ssp. spelta	Spelt	Esp 186	PI 348693
Triticum aestivum ssp. spelta	Spelt	Esp 188	PI 348696
Triticum aestivum ssp. spelta	Spelt	Esp 190	PI 348698
Triticum aestivum ssp. spelta	Spelt	Esp 192	PI 348701
Triticum aestivum ssp. spelta	Spelt	Esp 193	PI 348702
Triticum aestivum ssp. spelta	Spelt	Esp 196	PI 348712
Triticum aestivum ssp. spelta	Spelt	Esp 203	PI 348727
Triticum aestivum ssp. spelta	Spelt	Esp 204	PI 348728
Triticum aestivum ssp. spelta	Spelt	Esp 211	PI 348741
Triticum aestivum ssp. spelta	Spelt	Esp 216	PI 348747
Triticum aestivum ssp. spelta	Spelt	Esp 224	PI 348767
Triticum aestivum ssp. spelta	Spelt	Esp 227	PI 348771
Triticum aestivum ssp. spelta	Spelt	Esp 232	PI 469022
Triticum aestivum ssp. spelta	Spelt	Esp 233	PI 469023
Triticum aestivum ssp. spelta	Spelt	Esp 234	PI 469024
Triticum aestivum ssp. spelta	Spelt	Esp 236	PI 469026

Species	Common name	University of Cordoba Accession Number	Germplasm Bank Accession Number
Triticum aestivum ssp. spelta	Spelt	Esp 237	PI 469028
Triticum aestivum ssp. spelta	Spelt	Esp 238	PI 469029
Triticum aestivum ssp. spelta	Spelt	Esp 239	PI 469030
Triticum aestivum ssp. spelta	Spelt	Esp 240	PI 469031
Triticum aestivum ssp. spelta	Spelt	Esp 241	PI 469032
Triticum aestivum ssp. spelta	Spelt	Esp 242	PI 469042
Triticum aestivum ssp. spelta	Spelt	Esp 243	PI 469045
Triticum aestivum ssp. spelta	Spelt	Esp 244	PI 469046
Triticum aestivum ssp. spelta	Spelt	Esp 245	PI 469047
Triticum aestivum ssp. spelta	Spelt	Esp 246	PI 469048
Triticum aestivum ssp. spelta	Spelt	Esp 247	PI 469049
Triticum aestivum ssp. spelta	Spelt	Esp 248	PI 469050
Triticum aestivum ssp. spelta	Spelt	Esp 249	PI 469051
Triticum aestivum ssp. spelta	Spelt	Esp 250	PI 469053
Triticum aestivum ssp. spelta	Spelt	Esp 251	PI 469054
Triticum aestivum ssp. spelta	Spelt	Esp 252	PI 469056
Triticum aestivum ssp. spelta	Spelt	Esp 253	PI 469057
Triticum aestivum ssp. spelta	Spelt	Esp 254	PI 469058
Triticum aestivum ssp. spelta	Spelt	Esp 255	PI 469059
Triticum aestivum ssp. spelta	Spelt	Esp 256	PI 469060
Triticum aestivum ssp. spelta	Spelt	Esp 257	PI 469038
Triticum aestivum ssp. spelta	Spelt	Esp 258	PI 469039
Triticum aestivum ssp. spelta	Spelt	Esp 259	PI 469040
Triticum aestivum ssp. spelta	Spelt	Esp 260	PI 469034
Triticum aestivum ssp. spelta	Spelt	Esp 262	BGE 014252
Triticum aestivum ssp. spelta	Spelt	Esp 263	BGE 017153
Triticum aestivum ssp. spelta	Spelt	Esp 264	BGE 020898
Triticum aestivum ssp. spelta	Spelt	Esp 265	BEG 20903
Triticum aestivum ssp. spelta	Spelt	Esp 268	BGE 023734
Triticum aestivum ssp. spelta	Spelt	Esp 272	BGE 012920
Triticum aestivum ssp. spelta	Spelt	Esp 276	BGE 012937
Triticum aestivum ssp. spelta	Spelt	Esp 279	BGE 020935
Triticum aestivum ssp. spelta	Spelt	Esp 281	BGE 025420
Triticum aestivum ssp. spelta	Spelt	Esp 285	BGE 001972
Triticum aestivum ssp. spelta	Spelt	Esp 288	BGE 002005
Triticum aestivum ssp. spelta	Spelt	Esp 292	BGE 012932
Triticum aestivum ssp. spelta	Spelt	Esp 293	BGE 012906
Triticum aestivum ssp. spelta	Spelt	Esp 295	BGE 012935
Triticum aestivum ssp. spelta	Spelt	Esp 300	BGE 002006
Triticum aestivum ssp. spelta	Spelt	Esp 380	BGE 012903
Triticum aestivum ssp. spelta	Spelt	Esp 383	BGE 012902
Triticum aestivum ssp. spelta	Spelt	Esp 384	BEG 20900
Triticum aestivum ssp. spelta	Spelt	Esp 385	BGE 012931
Triticum aestivum ssp. spelta	Spelt	Esp 387	BGE 012766
Triticum aestivum ssp. spelta	Spelt	Esp 396	PI 469041

Species	Common name	University of Cordoba Accession Number	Germplasm Bank Accession Number
Triticum aestivum ssp. spelta	Spelt	Anna Maria	Anna Maria
Triticum aestivum ssp. aestivum	Common wheat	Antequera	Antequera
Triticum aestivum ssp. aestivum	Common wheat	Arthur Nick	Arthur Nick
Triticum aestivum ssp. aestivum	Common wheat	Conil	Conil
Triticum aestivum ssp. aestivum	Common wheat	Galera	Galera
Triticum aestivum ssp. aestivum	Common wheat	Montemayor	Montemayor
Triticum aestivum ssp. aestivum	Common wheat	Rota	Rota
Triticum aestivum ssp. aestivum	Common wheat	Santaella	Santaella
Triticum aestivum ssp. aestivum	Common wheat	Setenil	Setenil
Triticum aestivum ssp. aestivum	Common wheat	Tejada	Tejada

Table S4. Correlation coefficients among the evaluated traits in spelt.

	TW	TKW	GPC	Fe	Zn	PA	PA-Fe	PA-Zn	TOT-AX
TKW	-0.2995***								
GPC	-0.3532***	0.1477**							
Fe	-0.1372**	0.3246***	0.3402***						
Zn	-0.1955***	0.1658**	0.6389***	0.3847***					
PA	-0.2119***	0.0083 ns	0.2781***	0.0784 ns	0.4092***				
PA-Fe	-0.0153 ns	-0.2562***	-0.0960 ns	-0.7298***	-0.0466 ns	0.6101***			
PA-Zn	0.0408 ns	-0.1606**	-0.4243***	-0.3296***	-0.6894***	0.3636***	0.5266***		
TOT-AX	-0.1858***	0.1446**	0.1339*	-0.0005 ns	0.1180*	0.1413**	0.0900 ns	-0.0132 ns	
WE-AX	-0.3255***	0.2577***	0.1121*	0.0602 ns	0.1625**	0.2674***	0.1211*	0.0301 ns	0.3743***

***, **, *: significant at 99.9, 99 and 95%; ns: not significant.
